# A decision support system based on support vector machine for diagnosis of periodontal disease

**DOI:** 10.1186/s13104-020-05180-5

**Published:** 2020-07-13

**Authors:** Maryam Farhadian, Parisa Shokouhi, Parviz Torkzaban

**Affiliations:** 1 Department of Biostatistics, School of Public Health and Research Center for Health Sciences, Hamadan University of Medical Sciences, Hamadan, Iran; 2 Dental School, Hamadan University of Medical Sciences, Hamadan, Iran; 3 Department of Periodontics, Dental School, Dental Research Center, Hamadan University of Medical Sciences, P.O. Box 4171-65175, Hamadan, Iran

**Keywords:** Periodontics, Support vector machine, Classification, Diagnosis, Machine learning, Decision support systems

## Abstract

**Objective:**

Early diagnosis of many diseases is essential for their treatment. Furthermore, the existence of abundant and unknown variables makes more complicated decision making. For this reason, the diagnosis and classification of diseases using machine learning algorithms have attracted a lot of attention. Therefore, this study aimed to design a support vector machine (SVM) based decision-making support system to diagnosis various periodontal diseases. Data were collected from 300 patients referring to Periodontics department of Hamadan University of Medical Sciences, west of Iran. Among these patients, 160 were Gingivitis, 60 were localized periodontitis and 80 were generalized periodontitis. In the designed classification model, 11 variables such as age, sex, smoking, gingival index, plaque index and so on used as input and output variable show the individual’s status as a periodontal disease.

**Results:**

Using different kernel functions in the design of the SVM classification model showed that the radial kernel function with an overall correct classification accuracy of 88.7% and the overall hypervolume under the manifold (HUM) value was to 0.912 has the best performance. The results of the present study show that the designed classification model has an acceptable performance in predicting periodontitis.

## Introduction

Development of diagnostic systems that help physicians to diagnose and make more precise decisions in the treatment of these diseases has become commonplace. These medical decision support systems (MDSS) help physicians and professionals make more accurate decisions and reduce possible errors [1–3]. Different machine learning algorithms are often used in decision-making systems. One of the broad and widely used areas of artificial intelligence is machine learning, which studies the ways and algorithms that enable computers and systems to learn and to perform actions [4, 5]. Machine learning is related to topics such as data mining and statistics. Some machine learning applications include pattern recognition, data classification, and bioinformatics [4]. Powerful mathematical and statistical software has enabled the use of sophisticated approaches such as artificial neural networks and support vector machines (SVM) to predict and classify different outcomes [5, 6]. Using these tools can reduce the potential errors caused by fatigue or inexperience of clinical professionals in the diagnosis. Also, using these systems, the medical database can be analyzed in much less time and with further details.

As in all medical fields, successful dental treatment is possible only with the correct diagnosis of the disease. The diagnosis will be made by a bilateral examination of the clinical examination and radiography. In addition to these examinations, some other clinical examinations such as gingival index, plaque index, mobility index and clinical adhesion surface are used in the gum area [7–9]. However, these examinations may not be sufficient for a correct diagnosis.

In this regard, computer programs can help dentists to diagnose the disease as well as the correct path afterward. Along with the rapid development of computer technology, technology is still being developed in medicine, dentistry, and other medical fields [10, 11].

Periodontal disease is a common infectious disease in humans that is mainly caused by microbial plaque and various factors may affect or alter the manifestations of the disease. Although the potential impact of systemic disorders on periodontics has been proven, recent evidence suggests that periodontal infection may increase the likelihood of some systemic disorders or alter their natural course. Among the cases where the effects of periodontal infections have been proven, are cardiovascular disease (CHD) and complications of coronary heart disease such as angina pectoris and myocardial infarction [12, 13]. Given the high prevalence of periodontal diseases in Third World societies, prevention, identification, and early treatment can help prevent systemic diseases caused by it. The purpose of this system is to accelerate and facilitate diagnostic processes and to help dentists make accurate decisions.

## Main text

### Methods

In this cross-sectional study, the records of 300 patients referred to the Periodontics department of Hamadan University of Medical Sciences, west of Iran, between September 2016 and June 2018 were studied. Based on consultation with two experienced professionals of periodontology as well as a review of clinical studies, the desired variables were selected [14, 15, 21, 23]. The corresponding values of the selected variables were extracted and recorded for each patient with full confidentiality principles. This study was approved by the Research Ethics Committee of Hamadan University of Medical Sciences with IR.UMSHA.REC.1398.154.

#### Support vector machine

**Fig. 1:**
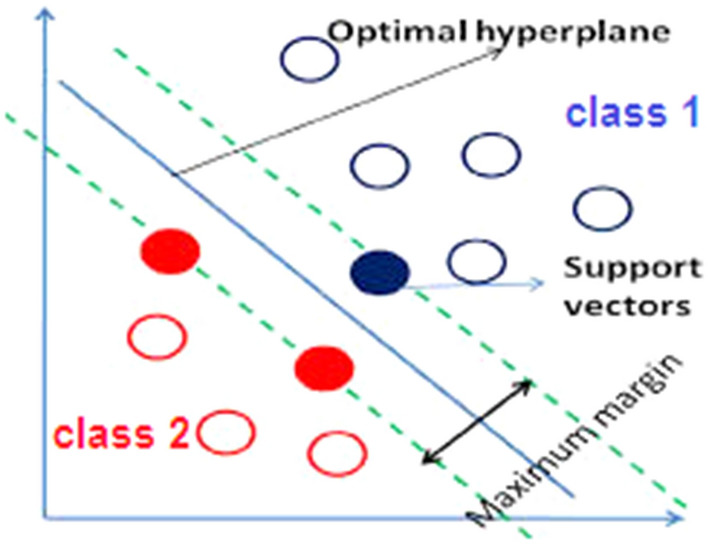
Support vector machine for linearity separable data

SVM is one of the supervised learning methods used for classification and regression. SVM is based on the theory of statistical learning. SVM is an algorithm that finds a particular type of linear model that maximizes the margin of hyper-planes. Maximizing the hyper-planes margin will maximize the separation between classes. The closest training points to the maximum cloud margin are the support vectors. Only these vectors (points) are used to specify the boundary between classes (Fig. 1). Assuming that the categories are linearly separable, it obtains the hyper planes with the maximum margin to separate the categories. The problem of finding the optimal line for the data is done by QP methods, which are well-known methods for solving constrained problems [16].

In problems where the data are not linearly separable, using a kernel function the data is mapped to a larger dimensional space so that it can be linearly separated in this new space. In this situation, various kernel functions, such as linear, radial, polynomials and sigmoid can be used [17].

#### Model performance evaluation

Cross-validation is a technique for assessing how the results of a predicting model such as SVM will generalize to an independent data set. The goal of cross-validation is to test the model’s ability to predict new data that was not used in constructing it.

One round of cross-validation involves splitting the entire data randomly into k subsets (folds), then the predictive model construct based on one subset (called the training set), and predictive performance evaluate based on the other subset (called the validation set or testing set). This process repeats until every K-fold serves as the test set. Finally, recorded accuracy measures combined (averages) to derive a more accurate estimate of model prediction performance [4].

Also, we try hypervolume under the ROC manifold (HUM) as an alternative error measure for evaluating the SVM model. Unlike the overall correct classification accuracy measure, HUM does not depend on the class prevalence and thus reflects the intrinsic accuracy of the classifier [18].

It should also be noted that in the current study the professional’s diagnosis is considered as the gold standard for evaluating the predictive performance of the model.

#### Building a SVM classification model

In SVM classification model, the variables including age (continues), sex (male, female), smoking (yes, no), attachment loss (continues), plaque index (percent), probing packet depth (continues), gingival index (grade I, grade II, grade III, grade IV), alveolar bone loss (score: 0 for ABL ≤ 20%, 1 for 20 ≤ ABL ≤ 50%, 2 for ABL ≥ 50% based on radiography), papilla bleeding index (0, 1, 2, 3), mobility (no, yes), and simplified oral hygiene index (0, 1, 2, 3) used as input and the individual’s status of periodontal disease as output variable consists of three class (gingivitis, localized periodontitis, generalized periodontitis). Also, different kernel functions such as linear kernel, polynomial, sigmoid, and radial evaluated. To evaluate the performance of the SVM model, the accuracy criterion and the configuration of the confusion matrix was used.

All analyses were performed by using “caret”, “HUM” and “mcca” a freely available package from the Comprehensive R (R3.6.3) Archive Network (CRAN).

### Results

Table 1 compares the mean of continuous variables related to attachment loss, plaque index, probing pocket depth and age in three groups. The results showed that the mean difference of attachment loss, plaque index, probing pocket depth variables in the three groups was significant, but the mean age was not significant in the three groups. Table 1 also shows the frequency distribution of variables such as gender, smoking, gingival index, papilla bleeding index, alveolar bone loss and oral health in groups. The results show that the groups are not sexually different. There was a significant difference between the three groups in all variables. In Table 2, a comparison of the performance of different SVM models based on different kernel functions in terms of the accuracy index and HUM values based on tenfold cross validation is presented. The results show that the SVM classification model based on radial kernel function has the best performance with an overall correct classification accuracy of 88.7% using tenfold cross-validation method. Also, category-specific correct classification for gingivitis was 96.0%, for localized periodontitis was 64.0%, and for generalized periodontitis was 92.2%. Further evaluation of the SVM model using HUM criteria showed the overall HUM value was equal to 0.912.

**Table 1:** Comparison of different variables between three classes of diseases (Hamadan in the west of Iran- September 2016 to June 2018)

Class				
Variable	Gingivitis	Localized periodontitis	Generalized periodontitis	P-value*
	Mean ± SD	Mean ± SD	Mean ± SD	
Age	32.91 ± 10.77	33.88 ± 10.52	34.23 ± 12.38	0.656
Attachment loss	1.63 ± 0.47	2.24 ± 0.61	3.39 ± 0.81	< 0.001
Plaque Index (%)	43.46 ± 17.82	62.13 ± 17.80	74.55 ± 15.09	< 0.001
Probing packet depth	1.48 ± 0.32	2.01 ± 0.54	3.25 ± 0.78	< 0.001

	n	%	n	%	n	%	P-value**
Sex							
Female	65	40.6	19	31.7	29	36.3	0.453
Male	95	59.4	41	68.3	51	63.8	
Smoking							
No	148	92.5	54	90	65	81.2	0.031
Yes	12	7.5	6	10	15	18.8	
Gingival Index							
Grade I	48	30	6	10	5	15	< 0.001
Grade II	78	48.8	24	40	27	33.8	
Grade III	31	19.4	26	43.3	39	43.8	
Grade IV	3	1.9	4	6.7	9	7.6	
Alveolar Bone Loss							
0	153	95.6	7	11.7	2	2.5	< 0.001
1	4	2.5	18	30.0	32	40.0	
2	3	1.9	35	58.3	46	57.5	
Papilla Bleeding Index							
0	41	25.6	8	13.3	9	11.3	< 0.001
1	78	48.8	22	36.7	13	16.3	
2	37	23.1	23	38.3	41	33.7	
3	4	2.5	7	11.7	17	9.3	
Mobility							
No	140	87.5	35	58.3	46	57.5	< 0.001
Yes	20	12.5	25	41.7	34	42.5	
Simplified Oral Hygiene Index							
0	5	3.1	9	15	10	12.5	< 0.001
1	16	10.0	16	26.7	11	13.8	
2	79	49.4	24	40.0	40	50.0	
3	60	37.5	11	18.3	19	23.8	

*ANOVA

**Chi Square Test

**Table 2:** Comparison of the performance of different kernel functions (tenfold cross validation)

Kernel	Accuracy	HUM value
Linear	0.817	0.831
Polynomial	0.811	0.839
*Radial*	*0.887*	*0.912*
Sigmoid	0.875	0.892

*HUM* hypervolume under the manifold

### Discussion

In this study, the SVM classification method was used to classify patients with periodontitis. The results of the present study show that the designed classification model has an acceptable performance in predicting periodontitis. Using a precise model to predict periodontal disease can be helpful to dentists with little experience. In fact, the use of such systems can lead to a decrease in fear (due to lack of knowledge, skills, being alone) and increased self-esteem, especially in young physicians. The development and evolution of these systems can provide the satisfaction of the stakeholders of health care systems and it is also possible that by design and using decision-making systems in physician assistant’s portable devices or medical computers in medical offices providing real-time medical tools to clinical practitioners to make more reliable diagnoses [19, 20].

Several studies confirmed that the prevalence and severity of periodontal disease increase with age [21]. A similar finding has been observed in the present study, although there was no statistically significant difference in mean age between different classes of the disease, the average age of patients increased with increasing severity of the disease.

There is accumulating evidence for a higher level of periodontal disease among smokers [21]. In the present study, the percentage of smokers increased with increasing severity of the disease.

In the following, the classification model presented in this study will be compared with some studies related to the prediction of periodontitis.

Ozden et al., conducted a study on the 150 periodontal patients. Three classification models including support vector machine, decision tree and neural network were used in this study. Among them, support vector machine and decision tree have higher accuracy for classification of periodontal disease, with 98% accuracy and the worst performance is the ANN with an accuracy of 46% [22].

Youssif et al., conducted a study on 30 patients based on clinical data including plaque index, pocket depth, and clinical attachment level as well as data from Hematoxylin and Eosin lam prepared from individuals which were divided into three diagnostic groups: gingival enlargement, chronic periodontitis and chronic gingivitis. The statistical model used is Feed Forward, Back propagation ANN with a precision rating of 100% [8].

Arbabi et al., conducted a study in Iran. They divided 190 patients into two groups (n = 160 for train) and (n = 30 for test) and studied them by examining age, sex, plaque index, probing pocket depth, and clinical attachment loss index as an input variables. Two Levenberg-Marquardet (LM) and Scaled Conjugate Gradient (SCG) algorithms have been used in this work, results showed that a better performance for the LM than SCG [23].

Papantonopoulos et al., used Artificial Neural Network Model (ANN) along with patients information’s to classifying patients in two categories of chronic periodontitis and aggressive periodontitis. The accuracy of ANN for classification of data was 90–98% [24].

In fact, there is no biological or computational reason why a particular classification method is better for predicting the result under different conditions. In general, it is not possible to find a method that is always the best for classifying different data sets. Therefore, more research is needed to find the best classifier for each data set.

In our opinion, more accurate results can be obtained when more patients with various types of persistent systemic and periodontal problems are added to the system. It is likely that the classification performances could be improved with the use of some other classifiers, future investigations can focus on different classification model such as fuzzy expert systems. Future studies can be performed to identify the most important predictive variables such as inflammatory markers, systemic factors, stress, and educational levels in the classification of periodontitis.

### Conclusions

The decision-making support system based on support vector machine was highly accurate for the diagnosis of periodontal disease. Designing accurate decision-making support systems facilitate and accelerate diagnostic processes. The system designed in this study will help less experienced dentists and young residents in making decisions for the diagnosis of periodontal disease.

## Limitations

Although from a practical point of view, the ability to repeat valid results in the real data is guaranteed by cross-validation method, however, any predictive model that uses professional’s diagnosis as a gold standard is prone to inherent uncertainty due to incorrect classification by whom, and this will remain not only in the present study but in all studies with this approach. However, the hope is that using the diagnosis of an experienced professional will be overcome this limitation.

## Data Availability

The datasets during and/or analyzed during the current study available from the corresponding author on reasonable request.

## Abbreviations

SVM: Support Vector Machine
MDSS: Medical Decision Support Systems
AL: Attachment Loss
PI: Plaque Index
PPD: Probing Packet Depth
GI: Gingival Index
ABL: Alveolar Bone Loss
PBI: Papilla Bleeding Index
MB: Mobility
